# Rush Medical College, Chicago

**Published:** 1872-03

**Authors:** 


### RUSH MEDICAL COLLEGE, CHICAGO.

The Spring Session for 1872 will begin Wednesday, March 6, and will continue to June 26—sixteen weeks.

The Rush Medical College building having been destroyed by the great fire, the Faculty, in order fully to maintain the interests of the College, and to preserve its advantages to students of medicine, have secured the lecture and clinic rooms of the Cook County Hospital, corner Eighteenth and Arnold streets, in which to hold the usual Spring Course. Having tested the convenience of these rooms during a large portion of the Winter Term, they feel assured that the instruction will be as thorough and satisfactory as heretofore; and, therefore, confidently recommend that students of every grade avail themselves of their advantages.

As a means of acquiring practical knowledge, the Spring Course offers superior inducements. The classes are comparatively small, and immediate personal inspection can be made of all experiments, operations and demonstrations, illustrating the several branches taught. The Hospitals, Dispensaries and Cliniques, filled to overflowing with patients, provide ample means for most thorough observation and instruction in Surgery, Obstetrics, Clinical Medicine, etc.

INSTRUCTORS.—Prof. J. H. Etheridge, M.D., Instructor in Principles and Practice of Medicine; C. D. Parkes, M.D., Instructor in Anatomy; F. L. Wadsworth, M.D., Instructor in Physiology; E. Fletcher Ingals, M.D., Instructor in Materia Medica; Curtis T. Fenn, M.D., Instructor in Obstetrics; J. E. Owens, M.D., Instructor in Diseases of the Urinary Organs; Prof. E. Powell, M.D., Instructor in Surgery; I. N. Danforth, M.D., Instructor in Pathological Histology; L. W. Case, M.D., Instructor in Chemistry; J. E. O'Brien, M.D., Instructor in Medical Philosophy; W. C. Hunt, M.D., Instructor in Microscopical Anatomy and Use of Microscope.

TERMS.—College Matriculation Ticket, $5 00. (This ticket must be obtained for the Spring Course, and will be good one year.) Hospital Ticket, $5 00.

For Circulars and other information, address the Secretary of the Spring Course, Curtis T. Fenn, M.D., 1155 Michigan Avenue.

